# Study protocol: multi-centre, randomised controlled clinical trial exploring stromal targeting in locally advanced pancreatic cancer; STARPAC2

**DOI:** 10.1186/s12885-024-13333-z

**Published:** 2025-01-20

**Authors:** Hemant M. Kocher, BCI-STARPAC2 team, Charlotte Ackermann, Garima Priyadarshini, Cheryl Lawrence, Nishtha Kotriwala, Ramona Georgescu, BPTB team, Rhiannon Roberts, Christine Hughes, Ahmet Imrali, Claude Chelala, Precision-Panc team, Judith Dixon-Hughes, David K. Chang, Peter Sasieni, Pippa Corrie, Mairéad G. McNamara, Debashis Sarker, Fieke E. M. Froeling, Alan Christie, Roopinder Gillmore, Khurum Khan, David Propper

**Affiliations:** 1 Barts Cancer Institute and Wolfson Institute of Public Health, Mary University of London, John Vane Science Centre, Charterhouse Square, London, Queen EC1M 6BQ UK; 2 Barts Health NHS Trust, Whitechapel, London, E1 1BB UK; 3 Cambridge University Hospitals NHS Foundation Trust, Cambridge, UK; 4 Division of Cancer Sciences, Department of Medical Oncology, University of Manchester &, The Christie NHS Foundation Trust, Manchester, UK; 5 Guy’s and St Thomas’ NHS Foundation Trust, London, UK; 6 University of Glasgowand, NHS Greater Glasgow and Clyde , Glasgow, UK; 7 Edinburgh Cancer Centre, NHS Lothian, Edinburgh, UK; 8 Royal Free London NHS Foundation Trust, London, UK; 9 University College London Hospitals NHS Foundation Trust, London, UK; 10 Centre for Tumour Biology, Queen Mary University of LondonBarts Cancer Institute– aCRUK Centre of Excellence, Charterhouse Square, London, EC1M 6BQ UK

**Keywords:** Locally advanced pancreatic ductal adenocarcinoma (laPDAC), Gemcitabine and nab-paclitaxel (GEM-NABP), All-trans-retinoic acid (ATRA), Chemotherapy, Stroma, Progression

## Abstract

**Background:**

Pancreatic cancer (PDAC: pancreatic ductal adenocarcinoma, the commonest form), a lethal disease, is best treated with surgical excision but is feasible in less than a fifth of patients. Around a third of patients presentlocally advanced, inoperable, non-metastatic (laPDAC), whose stadrd of care is palliative chemotherapy; a small minority are down-sized sufficiently to enable surgical excision. We propose a phase II clinical trial to test whether a combination of standard chemotherapy (gemcitabine & nab-Paclitaxel: GEM-NABP) and repurposing All Trans Retinoic Acid (ATRA) to target the stroma may extend progression-free survival and enable successful surgical resection for patients with laPDAC, since data from phase IB clinical trial demonstrate safety of GEM-NABP-ATRA combination to patients with advanced PDAC with potential therapeutic benefit.

**Methods:**

Patients with laPDAC will receive at least six cycles of GEM-NABP with 1:1 randomisation to receive this with or without ATRA to assess response, until progression or intolerance. Those with stable/responding disease may undergo surgical resection. Primary endpoint is progression free survival (PFS) defined as the time from the date of randomisation to the date of first documented tumour progression (response evaluation criteria in solid tumours [RECIST] v1.1) or death from any cause, whichever occurs first. Secondary endpoints include objective response rate (ORR), overall survival (OS), safety and tolerability, surgical resection rate, R0 surgical resection rate and patient reported outcome measures (PROMS) as measured by questionnaire EQ-5D-5L. Exploratory endpoints include a decrease or increase in CA19-9 and serum Vitamin A over time correlated with ORR, PFS, and OS.

**Discussion:**

STARPAC2 aims to assess the role of stromal targeting in laPDAC.

**Trial registration:**

EudraCT: 2019–004231-23; NCT04241276; ISRCTN11503604.

## Background

### Pancreatic cancer treatment options

Pancreatic ductal adenocarcinoma (PDAC) is a lethal disease. The only potentially curative procedure, surgical excision, is feasible in a minority of patients (~ 20%) [1]. Most patients present with advanced disease, with approximately 35% of patients having locally advanced (laPDAC), involving major vessels that precludes surgical excision, and approximately 45% are diagnosed with metastatic disease (mPDAC) [1]. Treatment options for patients with mPDAC and laPDAC remain minimally effective, with current options of 5-fluorouracil/leucovorin/irinotecan/oxaliplatin (FOLFIRINOX) or gemcitabine-nab-paclitaxel (GEM-NABP) giving an additional survival benefit of 4 and 2 months, respectively, when compared to gemcitabine monotherapy [2, 3].

Clinical trials (phase II or III) investigating treatment regimens for laPDAC include LAP-07 [4], SCALOP [5], LAPACT [6], NEOLAP [7] and SCALOP2 [8] to explore chemotherapy and chemoradiotherapy. Whilst chemotherapy gives the best results, radiotherapy in various forms or sensitizer agents after induction chemotherapy, hold promise which needs to be explored in further clinical trials. Thus, chemotherapy remains the cornerstone of laPDAC treatment with 5–30% undergoing surgical resection upon down-sizing of tumour. However, despite encouraging results, these regimens do not provide long-term survival benefit or good tumour control since the dense PDAC stroma acts as a barrier to chemotherapy delivery [9]. Thus, there is an urgent need for better therapeutic strategies.

Preclinical observations have demonstrated that all-trans retinoic acid (ATRA) is effective in reducing pancreatic stellate cell (key cell responsible for stromal barrier) proliferation by G1 cell-cycle arrest [10]. Standard chemotherapy when combined with ATRA, in various PDAC pre-clinical models, led to the restoration of physiological quiescent state of activated pancreatic stellate cells which in turn resulted in reduced proliferation, increased apoptosis and necrosis of cancer cells, increased immune cell infiltrate and alteration in tumour vasculature accompanied by collapse of the fibrotic stroma which together results in a substantial reduction of tumour size [11]. Thus, retinoids could potentially be used clinically as stromal targeting agents in combination with chemotherapy to reduce tumour size.

### Clinical studies of using retinoids in pancreatic cancer

All previous pancreatic cancer clinical trials used 13-cis retinoic acid, while STARPAC used ATRA. A phase II pilot trial of combining 13-cis RA with interferon-alpha (IFN-α) [12] demonstrated a median OS of 7.7 months (range, 0.9–23.9 + months) in histologically confirmed, unresectable PDAC (5 patients with laPDAC and 17 with mPDAC). In contrast, Moore et al. using the same combination, did not see any objective responses in six evaluable patients [13]. 13-cisRA (1 mg/kg on days 1–14) was combined with gemcitabine (1000 mg/m^2^ on days 8, 15, 22) for 20 evaluable patients with laPDAC and mPDAC and this demonstrated 1 partial remission, 7 with disease control and 12 with progressive disease [14].

All-trans retinoic acid (ATRA) alone or in combination with arsenic tri-oxide and / or induction chemotherapy has been established as standard treatment for patients with acute promyelocyctic leukaemia (APML) through a number of phase II/III clinical trials [15, 16]. The current recommended dose for ATRA is 45 mg/m^2^ for adults and children for this disease given in two equally divided doses and rounded to the nearest 10 mg increment.

STARPAC (stromal targeting in pancreatic cancer), a phase I clinical trial recruited 27 patients with mPDAC and laPDAC and determined the recommended phase 2 dose for nab-paclitaxel as 125 mg/m^2^ immediately followed by gemcitabine at 1000 mg/m^2^ on days 1, 8, 15 of a 28 day cycle with ATRA to be administered orally at 45 mg/m^2^ in two divided doses on days 1–15 of each cycle (recommended phase 2 dose (RP2D) and optimal biological dose (OBD)). There was no excess toxicity. There was a suggestion of efficacy that tumour shrinkage correlated with potential biomarkers such as CA19-9, Vitamin A levels, serum pentraxin 3 levels (PTX3) as well as tissue retinal binding proteins such as CRABP2 and FABP5 [17].

Thus, we sought to explore the additional benefits of the stromal targeting agent ATRA in combination with chemotherapy (GEM-NABP) for patients with laPDAC. We report the study protocol (version 7.0, 28th August 2024).

## Methods

### Study design and participants

**Fig. 1:**
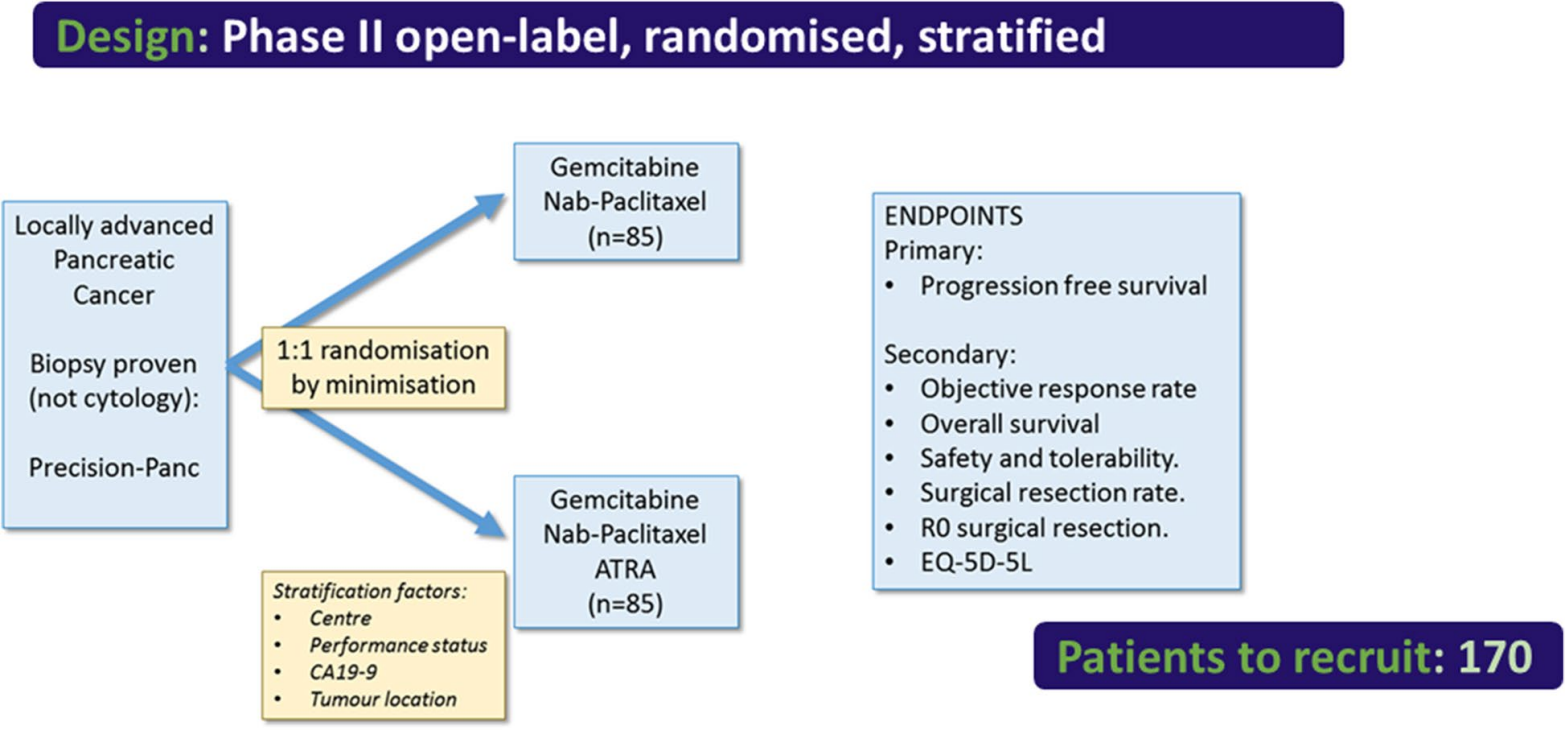
Schematic for STARPAC2. Abbreviations: ATRA = all-trans retinoic acid; EQ-5D-5L: European Quality of life, five dimensions, five levels

STARPAC2 is a multi-centre, open-label, stratified, randomised phase II clinical trial investigating safety and efficacy of GEM-NABP with or without ATRA in patients with laPDAC in the first-line setting (Fig. 1). Up to 16 UK centres are planned. Initial Ethical approval was granted by Research Ethics Committee (20/SC/0136) was granted on 29 May 2020. Study was registered NCT04241276 (24 January 2020); EudraCT: 2019–004231-23 (29 May 2020); ISRCTN11503604 (9th February 2021). Recruitment has opened in July 2020 with two stop-starts due to COVID19 and drug supply issues (March 2021- February 2022, Substantial amendments 2 and 3; August 2023 – August 2024, Substantial amendments 6 and 7: 28th August 24).

Entry criteria at registration include inoperable, non-metastatic, locally advanced PDAC with histological proof, Eastern Cooperative Oncology Group (ECOG) performance status of 0 or 1, and written informed consent (obtained at site via consent forms). After randomisation, chemotherapy (GEM-NABP) is administered with or without addition of ATRA at RP2D as determined by phase I trial data [17]. Table 1 lists the full entry criteria.

**Table 1:** Participant eligibility criteria for trial registration

Registration inclusion criteria	Registration exclusion criteria
1. Each patient must meet all of the following inclusion criteria to be enrolled in the study: 1. Written informed consent prior to admission to this study 2. Age ≥ 16 years. No upper age limit 3. ECOG performance status 0 or 1 4. Histologically proven pancreatic ductal adenocarcinoma (PDAC) as part of the Precision-Panc Master Protocol, or for patients who have gone exploratory laparotomy and found to have locally advanced disease 5. Locally advanced disease which is measurable according to the Response Evaluation Criteria in Solid Tumours (RECIST v1.1) 6. CT chest abdomen and pelvis as well as PET-CT within 28 days of randomisation (MRI Liver only if indeterminate liver lesions) to confirm absence of metastatic disease 7. Received no prior systemic therapy for pancreatic cancer 8. Adequate haematologic and end organ function, defined by the following laboratory results obtained within 14 days prior to the first study treatment: a. Absolute Neutrophil Count ≥ 1.5 × 10^9^/l (without granulocyte colony-stimulating factor support within 2 weeks prior to the first study treatment) b. Platelet count ≥ 100 × 10^9^/l (without transfusion within 2 weeks prior to the first study treatment) c. Haemoglobin ≥ 10 g/dl (transfusion permitted to establish target haemoglobin levels prior to the first study treatment) d. Calculated creatinine clearance (e.g. Cockcroft-Gault) ≥ 50 ml/min e. Bilirubin level ≤ 1.5 ULN (patients with known Gilbert disease who have bilirubin levels ≤ 3 × ULN may be enrolled). Patients must be able to undergo biliary stenting if required before or, if required, during the trial f. AST or ALT < 2.5 × ULN g. Alkaline phosphatase (ALP) < 2.5 × ULN h. INR and aPTT ≤ 1.5 × ULN; this applies only to patients who are not receiving therapeutic anticoagulation; patients receiving therapeutic anticoagulation should be on a stable dose 9. Female patients of child-bearing potential are eligible, provided they have a negative serum pregnancy test within 7 days prior to the first dose of study treatment, preferably as close to the first dose as possible. All patients with reproductive potential must agree to use a medically highly effective method of contraception throughout the treatment period and for 1 month after discontinuation of ATRA and / or gemcitabine/nab-paclitaxel (whichever is the latest) and for 6 months after discontinuation for male patients. Highly effective methods of contraception include IUD, oral contraceptive, transdermal implant. Micro-dosed progesterone preparations (“mini-pill”) are an inadequate method of contraception during treatment with ATRA. If patients are taking this pill they should be instructed to stop and another form of contraceptive should be prescribed instead 10. Able to follow protocol requirements as assessed by the Principal Investigator	A patient will not be eligible for inclusion in this study if any of the following criteria apply: 1. Patient has known metastases 2. Patient has experienced a significant reduction in performance status between the screening/ baseline visit and within 72 h prior to commencement of treatment as per trial protocol, and as per the Investigator’s assessment 3. Patients with pre-existing sensory neuropathy > grade 1 4. History of other malignancies (except cured basal or squamous cell carcinoma, superficial bladder cancer, prostate cancer in active surveillance, or carcinoma in situ of the cervix) unless documented free of cancer for ≥ 2 years 5. Patient has active, uncontrolled bacterial, viral, or fungal infection(s) requiring systemic therapy 6. Patient has known active, uncontrolled HIV, or active, uncontrolled hepatitis B or C infection. Patients with undetectable viral load are eligible 7. Patient has undergone major surgery, other than diagnostic surgery (i.e., surgery done to obtain a biopsy for diagnosis without removal of an organ), within 4 weeks prior to randomisation to this study 8. Patient has a history of allergy (including soya bean or peanut allergies) or hypersensitivity to any of the study drugs or any of their excipients, or the patient exhibits any of the events outlined in the Contraindications or Special Warnings and Precautions sections of the products or comparator SmPC or Prescribing Information 9. History of connective tissue disorders (e.g., lupus, scleroderma, arteritis nodosa) 10. Patient with a history of interstitial lung disease, history of slowly progressive dyspnoea and unproductive cough, sarcoidosis, silicosis, idiopathic pulmonary fibrosis, pulmonary hypersensitivity pneumonitis or multiple allergies 11. Patient with high cardiovascular risk, including, but not limited to, recent coronary stenting or myocardial infarction in the past year. A high cardiovascular risk is defined as person having recent (within last 12 months) coronary stenting or myocardial infarction or unstable angina which in the opinion of Principal Investigator, with or without a cardiologist opinion, is deemed to have an absolute risk for cardiovascular death of ≥ 5% over next 10 years. (ESC/ESH guidelines).^59^ 12. History of Peripheral Artery Disease (e.g., claudication, Leo-Buerger’s disease) 13. Patient has serious medical risk factors involving any of the major organ systems, or serious psychiatric disorders, which could compromise the patient’s safety or the study data integrity 14. Concurrent treatment with other experimental drugs or participation in another clinical trial with any investigational drug ≤ 30 days prior to study entry depending on the half-life of the investigational drug and/or guidance issued by the IMP manufacturer. Please contact the STARPAC2 coordinating team for further information 15. Patient is taking any prohibited concurrent medication, including vitamin A supplements, and is unwilling to stop use prior to and during the trial. Refer to Sect. 6.11.2 16. Patient is pregnant, planning to become pregnant or breast feeding Patient has received a live vaccine within four weeks prior to receiving their first dose of study treatment Patient is unwilling or unable to comply with study procedures, as assessed by the Principal Investigator

### Screening

Eligible patients are identified through Precision-Panc Master Protocol (NCT04161417, REC: 17/WS/0147), a UK national therapeutic development platform for pancreatic cancer. The Precision-Panc Master Protocol is sponsored University of Glasgow and NHS Greater Glasgow and Clyde and coordinated through Glasgow Oncology Clinical Trials Unit. The Precision-Panc Master Protocol allows consent of patients with PDAC to perform molecular profiling their tumour tissue and blood for, and then matches them to a series of PRIMUS (Pancreatic CanceR Individualised Multi-arm Umbrella Studies) including STARPAC2 (PRIMUS-005) if they have laPDAC. Exceptionally if patients have unresectable disease at laparotomy for resectable PDAC, they can be enrolled to STARPAC2 without entering Precision-Panc. All participants undergo a CT scan of the thorax, abdomen, and pelvis and a PET-CT scan (within 6 weeks before starting treatment, substantial amendment 4 to increase window from 4 to 6 weeks) as per NICE PDAC management guidelines (https://www.nice.org.uk/guidance/ng85). MRI liver can be conducted for indeterminate liver lesions and laparoscopy with laparoscopic ultrasound, for suspected small-volume peritoneal and/or liver metastases. The STARPAC2 Protocol uses a definition for laPDAC as currently updated in NCCN v3.0, July 2019 (https://www.nccn.org/professionals/physician_gls/pdf/pancreatic.pdf). A multi-disciplinary team should confirm absence of distant metastasis (liver, lung, peritoneum, bone, brain, distant lymph nodes, other) and unresectable local disease due to either solid contact with the superior mesenteric artery or coeliac artery for > 180º, or solid contact to the aorta, or unreconstructable superior mesenteric vein or portal vein or inferior vena cava due to tumour involvement, occlusion or thrombus (bland or tumour). CA19–9 and Vitamin A assessments are performed for all participants before treatment begins.

### Randomisation and stratification

After screening, participants eligible for STARPAC2 are randomised in a 1:1 ratio to one of two arms, using minimisation. Minimisation factors are centre location, WHO (World Health Organisation) performance status (0 or 1), disease location (head or body/tail) and CA19-9 levels. Randomisation is performed centrally by the Barts Centre for Experimental Medicine, using a computer-based algorithm. Patients also consent to use of residual samples and data to be donated to Barts Pancreas Tissue Bank (https://www.bartspancreastissuebank.org.uk/) for future research.

### Interventions

Gemcitabine and nab-paclitaxel chemotherapy with or without ATRA are the two arms of the trial. All registered patients aim to receive at least six cycles of gemcitabine and nab-paclitaxel (GEMNABP) chemotherapy, if tolerated: 125 mg/m^2^ nab-paclitaxel intravenously for 30 min, followed by 1000 mg/m^2^ gemcitabine intravenously for 30 min, both on day 1, 8, and 15 of a 28-day cycle. Those assigned to receive ATRA, receive this orally at 45 mg/m^2^ in two divided doses on days 1–15 of each cycle. Dose modifications and reductions are detailed in the Protocol, and are according to standard of care. Patients may withdraw participation at any time. Patients may be entered into follow-up treatment options at the end of six months, depending on the disease response status or continue on GEM-NABP: details are described in the Protocol.

### Post-treatment and on-treatment

Suitable patients are referred for surgery at any time during or after end of treatment. Participants are followed-up, after cessation of IMP treatment for every 12 weeks, with treatment-related toxicity, as per CTCAE V5.0, CA19-9, and assessment for disease status as appropriate at each visit.

Participants can discontinue if they withdraw consent at any time, if there is any medical condition that the Investigator or Sponsors Medical Assessor determines may jeopardise the participant’s safety if he or she continues in the study, if investigator or Sponsors Medical assessor determines it is in the best interest of the patient and if there is participant noncompliance.

Any study drug can be discontinued if any medical condition arises that may jeopardise the participant’s safety if they were to continue on study treatment in the judgment of the Investigator, if there is use of another systemic anti-cancer therapy, if there is pregnancy (for female participants only) or at participant request or due to protocol violations or non-compliance.

### On-trial assessment

Participants are assessed clinically prior to each cycle of chemotherapy to include weight, vital signs, ECOG performance status, and treatment-related toxicity, as per CTCAE V5.0 as well as EQ-5D-5L assessments. Haematology and biochemistry blood tests including vitamin A and CA19-9 measurements are undertaken ≤ 3 days before each subsequent cycle commencement, with further haematology and biochemistry as well as treatment-related toxicity assessment on days 8 and 15 of each cycle to ensure appropriate doses are administered. Outcome measurement assessments are discussed below.

### Safety reporting

Any adverse event (AE) that occurs from consent up to 30 days after the last treatment dose (safety visit) are collected. AEs are graded using the CTCAE V5.0 and data items include AE term, date of onset, date of resolution (or if ongoing), NCI CTCAE V5.0 grade (maximum intensity), seriousness, investigator causality rating against the study medication, action taken with regard to study medication, and outcome. Additional assessments will be carried out for ≥ grade 3 neuropathy or suspected pneumonitis. Serious adverse reactions (trial-treatment-related SAEs), will be collected until the end of follow-up. Suspected unexpected serious adverse reactions (SUSAR) will be reported to the Medicines and Health Care Product Regulatory Agency (MHRA).

### Endpoints and outcome measures

#### Primary outcome

The primary outcome measure is progression-free survival (PFS) measured as the time from the date of randomisation to the date of first documented tumour progression (response evaluation criteria in solid tumours [RECIST] v1.1) or death from any cause, whichever occurs first. The RECIST v1.1 guidelines are used for measurable, non-measurable, target lesions (TL), and non-target lesions (NTL) and the objective tumour response criteria (CR, PR, stable disease (SD), or progressive disease (PD) using baseline screening scan. Tumour assessments should be performed at screening (within 28 days prior to randomisation), every 8 weeks thereafter (± 7 days), and when clinically indicated for all patients. This schedule is to be maintained and will not be shifted for treatment delays. The same imaging method (triple phase CT scan of chest abdomen and pelvis is most commonly used) used to define disease sites at screening must be used throughout the study (e.g., the same contrast protocol for CT scans). Response assessments will be performed by the site Principal Investigator, on the basis of physical examinations and imaging scans and through use of RECIST v1.1. Patients who discontinue study treatment for any reason other than disease progression will continue to undergo tumour-response evaluations until progressive disease or initiation of other anti-cancer therapy.

### Secondary outcomes

Objective response rate (ORR) is defined as the number of patients with an objective response (OR) divided by the number of patients analysed. OR is defined as the number of patients with at least one confirmed response of complete response (CR) or partial response (PR). OR will be calculated in patients with measurable disease at baseline. Patients without measurable disease at baseline will not be included in this analysis. Patients without a post-baseline tumour assessment will be considered to be non-responders. Overall survival (OS) is defined as the time from the date of randomisation to death from any cause. All deaths will be included, whether they occur on study or following treatment discontinuation. For patients who have not died, OS will be censored at the date of last contact. Safety and tolerability will be assessed by AEs (CTCAE v5.0). Surgical resection is rate defined as patients undergoing complete resection of known pancreatic primary and associated lymph nodes at any point after enrolment, in each arm. R0 surgical resection rate is defined as histologically confirmed complete resection of the known pancreatic primary from those who undergo resection. Patient reported outcomes will be measured by the questionnaire EQ-5D-5L. Exploratory outcomes include a decrease or increase in CA19-9 serum Vitamin A over time which are correlated with ORR, PFS, and OS.

### Sample size

The sample size is based on the primary outcome measure. The estimated median PFS is 10 months for the control arm (gemcitabine + nab-paclitaxel). Using a group sequential design, this study is designed to have 90% power at the 20% one-sided level of statistical significance (equivalent to 80% power at the 10% one-sided level of statistical significance) to detect a 50% improvement in median PFS (i.e. an estimated hazard ratio of 0.667) leading to an estimated median PFS of 15 months. This design requires 170 patients (129 PFS events), randomised 1:1 between the two arms (85 patients in the control arm and 85 patients in the experimental arm), with no dropouts expected. It may be possible to conduct an informal assessment for futility at 43 PFS events (estimated at approximately 20 months)( using a Lan-DeMets spending function with a Pocock type boundary). If the observed hazard ratio is greater than 1.121 in favour of the control arm, the trial may be stopped for futility, if considered appropriate.

### Regulatory and monitoring committees

STARPAC2 is conducted in accordance with the regulatory requirements for clinical trials (MHRA), Research Ethics Committee (REC) approval and standard operating procedures of the Sponsor (Queen Mary University of London). Data management is via bespoke online database system, using electronic case report forms and anonymised data is stored confidentially at the Centre for Experimental Clinical Medicine at Barts Cancer Institute. There is also trial management group (TMG) and trial steering committee (TSC), with representation from medical oncologists, surgeons, trial management and statisticians, who will monitor and review the accumulated safety data and outcome measures.

### Translational research

Initial biopsies will be analysed as part of Precision-Panc Master Protocol. Blood samples and tumour biopsies are being collected for future translational work from all participants who consent to take part in the trial. All blood samples, data and biopsies (after assessment for Precision-Panc) will be stored at the Barts Pancreas Tissue Bank (REC Ref: 23/SC/0324, HTA Licensing number: 12199), Queen Mary University of London.

## Discussion

STARPAC2 is a natural evolution from the STARPAC phase 1 clinical trial to asess efficacy for stromal targeting in a group of patients with laPDAC with clinically meaningful primary and secondary outcome measures as well as safety monitoring. In addition, there is an opportunity to use biomaterial facilitated by prospective biobanking to develop clinically relevant biomarkers, novel therapies and mechanisms of resistance. STARPAC2 will also benefit from the Precision-Panc platform, facilitating genetic/molecular characterisation for the first time for laPDAC. laPDAC has not benefitted from sequencing, since most sequencing efforts have focused on easily accessible and plentiful material from rPDAC, or biopsy from mPDAC [18–20]. STARPAC2 also enriches laPDAC by using PET-CT to screen patients for metastatic disease for the first time in a clinical trial for laPDAC.

The trial results will be published in peer-reviewed journal(s) and presented at scientific conferences. For STARPAC2, management permission (“R&D approval”) was sought from all NHS organisations involved in the study, in accordance with NHS research governance arrangements, as listed here: Barts Health NHS Trust; Cambridge University Hospitals NHS Foundation Trust; The Christie NHS Foundation Trust; Guy’s and St Thomas’ NHS Foundation Trust; NHS Greater Glasgow and Clyde; NHS Lothian; Royal Free London NHS Foundation Trust; University College London Hospitals NHS Foundation Trust. Further sites will open in due course. STARPAC2 is open to international collaboration. Interested investigators are invited to contact the trials unit (bci-starpac2@qmul.ac.uk) for further discussion.

## Data Availability

No datasets were generated or analysed during the current study.

## Abbreviations

AE: Adverse event
APML: Acute promyelocyctic leukemia
ATRA: All-trans retinoic acid
brPDAC: Borderline resectable pancreatic ductal adenocarcinoma
CT: Computerised tomography
CTCAE: Common Toxicity Criteria For Adverse Events
ECOG: Eastern cooperative oncology group
FFPE: Formalin fixed paraffin embedded
HTA: Human Tissue Act
laPDAC: Locally advanced pancreatic ductal adenocarcinoma
mPDAC: Metastatic disease pancreatic ductal adenocarcinoma
MRC: Medical Research Council
MRI: Magnetic resonance imaging
NHS: National Health Service
NICE: National Institute for Health and Care Excellence
NTL: Non-target lesion
OBD: Optimal biological dose
ORR: Objective response rate
OS: Overall survival
PDAC: Pancreatic ductal adenocarcinoma
PET: Positron emission tomography
PFS: Progression free survival
PI: Principal investigator
PRIMUS: Pancreatic Cancer Individualized Multi-arm Umbrella Study
PSC: Pancreatic stellate cells
PTX3: Serum Pentraxin 3
Queen Mary: Queen Mary University of London
QOL: Quality of life
R0: Resection margin negative
RA: Retinoic acid
REC: Research ethics committee
RECIST: Response Evaluation Criteria In Solid Tumours
RP2D: Recommended Phase 2 Dose
rPDAC: Resectable pancreatic ductal adenocarcinoma
RR: Response rate
SAE: Serious adverse event
SAR: Serious Adverse Reaction
SD: Stable disease
SUSAR: Suspected Unexpected Serious Adverse Reaction
TL: Target lesion
TMG: Trial management group
TSC: Trial steering committee
